# Characterisation of (*R*)-2-(2-Fluorobiphenyl-4-yl)-*N*-(3-Methylpyridin-2-yl)Propanamide as a Dual Fatty Acid Amide Hydrolase: Cyclooxygenase Inhibitor

**DOI:** 10.1371/journal.pone.0139212

**Published:** 2015-09-25

**Authors:** Sandra Gouveia-Figueira, Jessica Karlsson, Alessandro Deplano, Sanaz Hashemian, Mona Svensson, Marcus Fredriksson Sundbom, Cenzo Congiu, Valentina Onnis, Christopher J. Fowler

**Affiliations:** 1 Department of Pharmacology and Clinical Neuroscience, Pharmacology Unit, Umeå University, Umeå, Sweden; 2 Department of Chemistry, Umeå University, Umeå, Sweden; 3 Department of Life and Environmental Sciences, Unit of Pharmaceutical, Pharmacological and Nutraceutical Sciences, University of Cagliari, via Ospedale 72, Cagliari, Italy; University of Parma, ITALY

## Abstract

**Background:**

Increased endocannabinoid tonus by dual-action fatty acid amide hydrolase (FAAH) and substrate selective cyclooxygenase (COX-2) inhibitors is a promising approach for pain-relief. One such compound with this profile is 2-(2-fluorobiphenyl-4-yl)-*N*-(3-methylpyridin-2-yl)propanamide (Flu-AM1). These activities are shown by Flu-AM1 racemate, but it is not known whether its two single enantiomers behave differently, as is the case towards COX-2 for the parent flurbiprofen enantiomers. Further, the effects of the compound upon COX-2-derived lipids in intact cells are not known.

**Methodology/Principal Findings:**

COX inhibition was determined using an oxygraphic method with arachidonic acid and 2-arachidonoylglycerol (2-AG) as substrates. FAAH was assayed in mouse brain homogenates using anandamide (AEA) as substrate. Lipidomic analysis was conducted in unstimulated and lipopolysaccharide + interferon γ- stimulated RAW 264.7 macrophage cells. Both enantiomers inhibited COX-2 in a substrate-selective and time-dependent manner, with IC_50_ values in the absence of a preincubation phase of: (*R*)-Flu-AM1, COX-1 (arachidonic acid) 6 μM; COX-2 (arachidonic acid) 20 μM; COX-2 (2-AG) 1 μM; (*S*)-Flu-AM1, COX-1 (arachidonic acid) 3 μM; COX-2 (arachidonic acid) 10 μM; COX-2 (2-AG) 0.7 μM. The compounds showed no enantiomeric selectivity in their FAAH inhibitory properties. (*R*)-Flu-AM1 (10 μM) greatly inhibited the production of prostaglandin D_2_ and E_2_ in both unstimulated and lipopolysaccharide + interferon γ- stimulated RAW 264.7 macrophage cells. Levels of 2-AG were not affected either by (*R*)-Flu-AM1 or by 10 μM flurbiprofen, either alone or in combination with the FAAH inhibitor URB597 (1 μM).

**Conclusions/Significance:**

Both enantiomers of Flu-AM1 are more potent inhibitors of 2-AG compared to arachidonic acid oxygenation by COX-2. Inhibition of COX in lipopolysaccharide + interferon γ- stimulated RAW 264.7 cells is insufficient to affect 2-AG levels despite the large induction of COX-2 produced by this treatment.

## Introduction

According to the textbooks, non-steroidal anti-inflammatory drugs (NSAIDs) produce their effects upon pain and inflammation as a result of the inhibition of cyclooxygenase (COX)-derived prostaglandin production [1]. There is, however, evidence that NSAIDs also involve the endocannabinoid (eCB) system in their actions. Thus, for example, the effects of spinal administration of the NSAID indomethacin in the formalin test of prolonged pain is blocked by a CB_1_ receptor antagonist and is not seen in CB_1_^-/-^ mice [2], and similar effects have been seen with other spinally administered NSAIDs (review, see [3]). In 2010, Bishay et al. [4] reported that the (*R*)-enantiomer of flurbiprofen produced CB receptor-mediated effects in a model of neuropathic pain. Given that the COX-inhibitory properties of the profens such as flurbiprofen are traditionally considered to reside in the (*S*)-enantiomer [5], this is an important result and may have bearing upon the analgesic properties of this compound.

The two most well-studied endogenous ligands for the eCB system are anandamide (AEA) and 2-arachidonoylglycerol (2-AG). AEA and 2-AG are effectively metabolised both by hydrolytic and other pathways [6]. With respect to the former, AEA is hydrolysed by both fatty acid amide hydrolase (FAAH) and *N*-acylethanolamine acid amidase (NAAA), whilst 2-AG is hydrolysed by monoacylglycerol lipase, α/β-hydrolase domain 6/12 and FAAH to form arachidonic acid [6]. However, AEA and 2-AG are also substrates for COX-2 [7,8]. In 2009, Prusakiewicz et al. [9] reported that ibuprofen and mefenamic acid were more potent inhibitors of the oxygenation of 2-AG by COX-2 than of the oxygenation of arachidonic acid by this enzyme isoform. COX-2 is a homodimeric enzyme, and the authors suggested that the selectivity was due to the fact that 2-AG oxygenation was blocked when the inhibitor had bound to one of the monomers, whereas blockade of arachidonic acid metabolism required binding to both monomers [9]. This type of substrate-selective inhibition may be therapeutically useful: an indomethacin analogue, LM-4131, has also been identified as a substrate-selective inhibitor of COX-2. The compound increases brain 2-AG levels, and produces potentially beneficial effects in models of anxiety [10]. This group has also investigated the (*R*)-enantiomers of the profens and found them to be potent inhibitors of AEA and 2-AG oxygenation without affecting arachidonic acid oxidation [11]. They showed further that in dorsal root ganglion cells cultured under inflammatory conditions, the (*R*)-enantiomers of ibuprofen, naproxen and flurbiprofen increased AEA and 2-AG levels without affecting arachidonic acid levels [11]. It is not known, however, whether substrate-selective COX-2 inhibitors increase AEA and 2-AG in other cells, such as macrophages, when they are cultured under inflammatory conditions.

An important unwanted property of NSAIDs is their propensity to cause gastric lesions. In a key study, Naidu et al. [12] showed that not only did the FAAH inhibitor URB597 reduce the gastrointestinal damage produced by the NSAID diclofenac, but also acted synergistically with this compound in a model of visceral pain. This and other findings has led to the suggestion that dual-action FAAH-COX inhibitors may be useful for the treatment of pain [13]. Recently, Sasso et al. [14] reported the synthesis of a compound, ARN2508. The compound inhibited FAAH and both COX isoforms in an irreversible manner and reduced concentrations in the plasma of the prostaglandin metabolite 6-keto PGF_1α_ whilst increasing levels of the FAAH substrates PEA and OEA. ARN2508 showed efficacy in a model of inflammatory pain without producing gastric lesions [14]. It is not known, however, whether the compound inhibits COX-2 in a substrate-selective manner.

In 2003, two of us (C.C., V.O.) reported that the amide derivative of ibuprofen with 2*-*amino-3-methylpyridine was more potent than ibuprofen in a model of visceral pain, but had a considerably lower ulcerogenic potency [15]. This compound was subsequently shown to inhibit FAAH with a potency approximately 2–3 orders of magnitude greater than ibuprofen itself, whilst retaining its COX-inhibitory properties [16,17]. We have also reported that the corresponding amide analogue of flurbiprofen (Flu-AM1) is a potent inhibitor of rat brain FAAH and additionally shows a substrate-selective inhibition of COX-2, whereby lower concentrations were needed to block the oxygenation of 2-AG than of arachidonic acid [18]. As with flurbiprofen, Flu-AM1 has a chiral centre. In view of the clear enantiomeric difference seen with flurbiprofen towards the inhibition of COX-2 [11], it is important to investigate whether the profile of Flu-AM1 as a dual action FAAH: substrate-selective COX-2 inhibitor can be further refined by selection of the (*R*)-enantiomer.

Thus from the above discussion, two distinct questions can be formulated:

- Do the amide derivatives of flurbiprofen with 2-amino-3-methylpyridine show enantiomeric differences with respect to inhibition of COX-2 and FAAH?
- Is COX-2 inhibition *per se* sufficient to affect endocannabinoid levels in macrophage cells cultured under inflammatory conditions?

These questions have been investigated in the present study.

## Materials and Methods

### Compounds and materials

Radioactive arachidonoyl ethanolamide[1-^3^H] ([^3^H]-AEA) was obtained from American Radiolabeled Chemicals, Inc (St Louis, MO, USA). (*R*)(-)-Flurbiprofen and (*S*)(+)-Flurbiprofen were purchased from Santa Cruz Biotechnology Inc. (Dallas, Texas, USA). Ovine COX-1 (cat. no. 60100), human recombinant COX-2 (cat. no. 60122), COX-2 polyclonal antibody (rabbit anti-mouse, cat #: 160106), arachidonic acid, 2-AG, AEA and URB597 (cyclohexylcarbamic acid 3′-carbamoylbiphenyl-3-yl ester) were purchased from the Cayman Chemical Co. (Ann Arbor, MI, USA). Substrates were dissolved in ethanol or DMSO as appropriate. Polyclonal goat anti-rabbit immunoglobulin/HRP was obtained from Dako (Glostrup, Denmark). Protease inhibitor cocktail set III was obtained from Merck Millipore (Darmstadt, Germany). For the lipid quantification experiments, the following native and deuterated standards were purchased from the Cayman Chemical Co.: AEA, 2-AG, PEA, OEA, DEA, LEA, SEA, 2-LG, AEA-d^8^, 2-AG-d^8^, PEA-d^4^, SEA-d^3^, OEA-d^4^, PGF_2α_, PGE_2_, TXB_2_, PGD_2_, 12(13)-EpOME, 9(10)-DiHOME, 12(13)-DiHOME, 11-HETE, 12-HETE, 15-HETE, 9-HODE, 13-HODE, 12-HEPE, 12-[[(cyclohexylamino)carbonyl]amino]-dodecanoic acid (CUDA), 12(13)-DiHOME-d^4^, 12(13)-EpOME-d^4^, 9-HODE-d^4^, PGE_2_-d^4^ and PGD_2_-d^4^. 9,10,13-TriHOME and 9,12,13-TriHOME were obtained from Larodan (Sweden, Malmö). For list of lipid abbreviations, see S1 Table.

### Synthesis of the enantiomers of Flu-AM1

Enantiomerically pure (*R*)(-)-Flu-AM1 and (*S*)(+)-Flu-AM1were synthesized using a slight modification of the procedure previously described for the racemate [18]. Briefly, commercially available (*R*)(-)-Flurbiprofen and (*S*)(+)-Flurbiprofen (0.24 g, 1 mmol) in dry acetonitrile solution (10 mL) were added with 1-(3-dimethylaminopropyl)-3-ethylcarbodiimide hydrochloride (0.21 g, 1.1 mmol) and hydroxybenzotriazole (0.13 g, 1 mmol). This mixture was stirred at room temperature for 30 min and then treated with 2-amino-3-methylpyridine (0,11 g, 1 mmol), after which the mixture was stirred at room temperature for additional 24 h. Solutions were evaporated to dryness *in vacuo* and the residues were dissolved in ethyl acetate (20 mL) and washed sequentially with brine (2 x 5 mL), 10% aqueous sodium carbonate (2 x 5 mL), 10% aqueous citric acid (2 x 5 mL), and water (2 x 5 mL). The organic layer was dried over anhydrous magnesium sulphate, filtered, and concentrated to dryness under reduced pressure. The respective (*R*)(+)-Flu-AM1 or (*S*)(-)-Flu-AM1 was obtained (54 and 52% yield respectively) in analytically pure form. ^1^H NMR (DMSO-d_6_, recorded on a Varian Inova 500 spectrometer in DMSO-d_6_ solution, with chemical shifts (*δ*) given in part per million downfield from the internal standard, tetramethylsilane): *δ* 1.42 (d, *J* = 7.0 Hz, 3H, CH_3_), 2.10 (s, 3H, CH_3_), 3.91 (q, *J* = 7.0 Hz, 1H, CH), 7.21–7.52 (m, 10H, Ar and Py), 7.98 (s, 1H, Ar), 10.03 (s, 1H, NH). Infrared spectra (recorded on a Bruker Vector 22 spectrometer in Nujol mull): 3330, 3020, 2965, 1675, 1638, 1576 cm^-1^. Optical rotation (assessed at 10 mg/mL concentrations using a Perkin Elmer 241 polarimeter in a 10 cm water-jacketed cell at 25°C): [α] = -11.2° for (*R*)(-)-Flu-AM1 and [α] = +11.5° for (*S*)(+)-Flu-AM1. MS (positive-ion electrospray ionization (ESI) mass spectra recorded on a double-focusing Finnigan MAT 95 instrument with BE geometry): *m/z* 335 (M + H)^+^. Combustion elemental analyses (conducted with a Yanagimoto MT-5 CHN recorder elemental analyzer): Anal. Calcd. for C_21_H_19_FN_2_O: C, 75.43; H, 5.73; N, 8.83. Found: C, 75.47; H, 5.72; N, 8.89 for (*R*)(-)-Flu-AM1 and C, 75.37; H, 5.75; N, 8.90 for (*S*)(+)-Flu-AM1.

### COX-1 and 2 inhibition experiments

The assay was performed according to Meade *et al*. [19] with minor modifications [20]. An oxygen electrode chamber with integral stirring (Oxygraph System, Hansatech Instruments, King´s Lynn, U.K.) was calibrated daily to ambient temperature and air pressure. The assay buffer contained 0.1 M Tris-HCl buffer pH 7.4, 1 μM haematin, 2 mM phenol, 5 mM EDTA, 10 μM substrate (arachidonic acid or 2-AG) (final assay volume was 2 ml). After addition of (*R*)- or (*S*)-Flu-AM1 dissolved in ethanol (final assay concentration 1%), a baseline was established for 5 min before initiation of reaction by addition of 200 units ovine COX-1 or human recombinant COX-2. The change in oxygen consumption as a measurement of enzyme activity was monitored for approximately 5 min.

### FAAH assay

Brains from male B6CBAF1/J mice, stored at -80°C, were thawed, weighed and homogenized in cold buffer (20 mM HEPES, 1 mM MgCl_2_ pH 7.0). Homogenates were centrifuged (35,000 g at 4°C for 20 min) before the pellet was resuspended in cold homogenization buffer. Centrifugation and resuspension was repeated twice. The suspension was incubated at 37°C for 15 min to degrade any endogenous substrate able to interfere with the FAAH assay. After centrifugation (35,000 g at 4°C for 20 min), the pellet was resuspended in cold buffer (50 mM Tris-HCl, 1mM EDTA, 3 mM MgCl_2_, pH 7.4). The protein concentration was determined according to the Bradford assay [21] after which the samples were frozen in aliquots at -80°C. Ethical permission for the animal experiments was obtained from the local animal research ethical committee (Umeå Ethical Committee for Animal Research, Umeå, Sweden). FAAH was assayed using the method of Boldrup et al. [22], whereby homogenates (0.3 μg protein / assay) in assay buffer (10 mM Tris-HCl, 1mM EDTA, pH 7.4), test compounds and substrate ([^3^H]AEA in 10 mM Tris-HCl, 1mM EDTA, pH 7.4 containing 10 mg/ml fatty acid-free bovine serum, assay concentration 0.5 μM unless otherwise stated) were incubated for 10 min at 37°C. Thereafter, 400 μL activated charcoal (80 μL activated charcoal + 320 μL 0.5 M HCl) was added and the samples were placed on ice and centrifuged at 2500 rpm for 10 min. Aliquots (200 μL) of the supernatant was analyzed for tritium content by liquid scintillation spectroscopy with quench correction.

### LPS/INF-γ treatment of RAW 264.7 cells

RAW264.7 mouse leukemic monocyte/macrophage cells (European collection of cell cultures, Port Down, UK) were cultured in DMEM medium containing 4 mM glutamine, 10% foetal bovine serum and 100 U/mL penicillin and 100 μg/mL streptomycin. The cells were cultured in a 75 cm^2^ flasks at 37°C with 5% CO_2_ at humidified atmospheric pressure and split (ratio 1:3–6) approximately twice a week. LPS/INF-γ treatment was undertaken on RAW 264.7 cells seeded in 6 well plates. For the lipid measurements, 2.5 x 10^5^ cells/well were seeded and either phosphate-buffered saline (unstimulated) or LPS (0.1 μg/mL) + INF-γ (100 U/mL) were added immediately and the cells cultured for 24 h. Medium was then discarded. Test compounds (1 μM URB597, 10 μM flurbiprofen, 1 μM URB597 + 10 μM flurbiprofen, 10 μM (*R*)-Flu-AM1 or vehicle) were added, and the cells were incubated for 30 min at 37°C. When indicated, the calcium ionophore ionomycin (5 μM) was added at the same time as the test compounds. The plates were placed on ice and after removal of medium, the cells were washed twice with ice-cold PBS (2x1mL). One mL of methanol was added to the wells and the mixture was scraped using a rubber policeman and the extract pipetted into Falcon tubes. An additional 1 mL of methanol was added to the wells, the wells were scraped and the mixture was pipetted into the same tubes. These were then centrifuged at 2000 x g for 15 min (4°C) to sediment cell debris, and the methanol phase collected and stored at -80°C until used for analysis of prostaglandins, 2-AG, AEA and related lipids.

### [^3^H]AEA hydrolysis by intact RAW 264.7 cells

For the studies of [^3^H]AEA hydrolytic capacity of RAW 264.7 cells, initial experiments indicated that low activities were seen. In consequence, 2.5 x 10^6^ cells/well were seeded and cultured overnight prior to addition of either phosphate-buffered saline or LPS (0.1 μg/mL) + INF-γ (100 U/mL) and incubation for a further 24 h. [^3^H]AEA hydrolytic capacity was then measured as described previously [23]. Briefly, the cells were washed twice with 400 μL of pre-warmed KRH buffer (120mM NaCl, 4.7mM KCl, 2.2 mM CaCl_2_, 10mM 4-(2-hydroxyethyl)-piperazineethane-sulfonic acid (HEPES), 0.12 mM KH_2_PO_4_, 0.12 mM MgSO_4_, pH 7.4) with 1% BSA prior to addition of 340 μL of pre-warmed KRH buffer with 0.1% fatty acid-free BSA and 10 μL of test compound (final concentrations as above) or vehicle (0.05% DMSO + 0.1% ethanol). After preincubation for 10 min at 37°C, [^3^H]AEA (50 μL, final concentration 0.1 μM, in KRH buffer with 0.1% fatty acid-free BSA) was added and the cells were incubated for a further 60 min at 37°C. Reactions were stopped by addition of 600 μL of activated charcoal buffer (120 μL activated charcoal + 480 μL 0.5 M HCl) and the samples were then worked up as described above for the FAAH assay.

### Western blot for COX-2

RAW 264.7 cells (2.5 x 10^6^ cells/well) were seeded into 6 well plate and incubated for 24 h at 37°C prior to treatment with either phosphate-buffered saline or LPS (0.1 μg/mL) + INF-γ (100 U/mL). Following incubation for 1.5–24 h at 37°C, medium was aspirated, 400 μl ice-cold phosphate-buffered saline was added, and the cells were scraped using a rubber policeman. This procedure was repeated, and the samples were centrifuged for 4 min at 1000 r.p.m., 4°C to sediment the cells. A mixture of 150mM NaCl, 50mM Tris, 1% Triton-X100, pH 8.0 + Protease Inhibitor III (1:200 v.v^-1^, 500 μL) was added to the cells in Eppendorf tubes, which were then shaken for 30 min at 750 r.p.m., 4°C. Samples were then centrifuged at 14,000 g for 5 min at 4°C, and the supernatants then frozen at -80°C until used. Proteins in samples (20 μL, containing 3 μg of the samples and 1 x Laemmli buffer) were separated by gel electrophoresis using Mini Protean TGX stain free gels (BioRad, Hercules, CA, USA, cat # 456–8093; 200 V x 35 min). Human recombinant COX-2 (750 ng) was used as positive control. Proteins were transferred to PVDF mini membranes using a Trans-Blot Turbo Transfer System (BioRad). The membranes were treated with blocking solution (5% dried milk in 1 x tris-Buffered saline / Tween-20 [TBST] solution, 1 hour at room temperature) after which the primary antibody (COX-2 polyclonal antibody, rabbit anti-mouse, 1:1000, in 5% dried milk / TBST) was added and the membranes were left overnight at 4°C on a rotating table. After five washes with TBST, the membranes were treated with the secondary antibody (HRP-conjugated goat anti-rabbit, 1:2000) for 1 h at room temperature on a rotating table. After five washes, the membranes were treated with Clarity Western ECL substrate and photographed in a Molecular Imager Gel Doc XR system (BioRad) and quantified using ImageLab software 5.1 according to the manufacturers instructions (http://www.bio-rad.com/webroot/web/pdf/lsr/literature/Bulletin_6434.pdf).

### Cell viability experiments

RAW 264.7 cells (2.5 x 10^6^ cells/well) were seeded into 6 well plate and incubated for 24 h at 37°C prior to treatment with either phosphate-buffered saline or LPS (0.1 μg/mL) + INF-γ (100 U/mL). Following incubation for 1.5–24 h at 37°C, medium was aspirated, 400 μL ice-cold phosphate-buffered saline was added, and the cells were scraped using a rubber policeman. Cell viability was assessed using trypan blue and a TC20 automated cell counter (Bio-Rad).

### Assay of prostaglandins, 2-AG, AEA and related lipids in extracts from RAW 264.7 cells

Cell extracts were thawed on ice and milliQ water was added to give a final methanol concentration of 5% (v/v). After samples were spiked with 10 μL internal standard solutions (800 ng/mL 2-AG-d_8_, 40 ng/mL PGF_2α_-EA-d4 and PGE_2_-EA-d4, 20 ng/mL AEA-d_4_ and OEA-d_4_, PEA-d_4_ and SEA-d_3_, 50 ng/mL 12(13)-DiHOME-d_4_ and 12(13)-EpOME-d_4_ and 25 ng/mL 20-HETE-d_6_, 5(S)-HETE-d_8_, 9(S)-HODE-d_4_, PGE_2_-d_4_, PGD_2_-d_4_ and TXB_2_-d_4_, 10 μL antioxidant solution (0.2 mg/mL BHT/EDTA in methanol/water (1:1)) and then applied directly to the solid phase extraction cartridge. Briefly, compounds were extracted using Waters Oasis HLB cartridges (60 mg of sorbent, 30 μm particle size). Cartridges were washed with 2 mL of ethyl acetate, followed by 2x2 mL of MeOH, and then conditioned with 2x2 mL of wash solution (95:5 v/v water/methanol with 0.1% acetic acid). After loading the sample containing internal standard and antioxidant solution, the cartridges were washed with 2x4 mL of wash solution, dried under high vacuum for about 1 minute, and eluted with 3 mL acetonitrile, followed by 2 mL of methanol and 1 mL of ethyl acetate into polypropylene tubes containing 6 μL of a glycerol solution (30% in methanol). Eluates were concentrated with a MiniVac system (Farmingdale, NY, U.S.A.) and reconstituted in 100 μL of methanol and vortexed. If necessary, samples were centrifuged to remove any residuals. Solutions were then transferred to LC vials with low-volume inserts, 10 μL of a recovery standard (CUDA, 50 ng/mL) was added, to normalise for changes in volume and instrument variability, and UPLC-MS/MS analysis was performed immediately.

Chromatographic separation of the analytes was performed using an Agilent ultra-performance (UP)LC system (Infinity 1290) was coupled with an electrospray ionization source (ESI) to an Agilent 6490 Triple Quadrupole system equipped with the iFunnel Technology (Agilent Technologies, Santa Clara, CA, USA) [24]. Separate injections for subsequent ionization in positive (for 2-AG, AEA and related *N*-acylethanolamines) and negative mode (for the prostaglandins and other oxylipins) were undertaken. Analyte separation was performed using a Waters BEH C18 column (2.1 mm x 150 mm, 2.5 μm particle size), and 10 μL injection volumes were employed for each run. The mobile phase consisted of (A) 0.1% acetic acid in MilliQ water and (B) acetonitrile:isopropanol (90:10). The following gradients were employed: 0.0–3.5 min 10–35% B, 3.5–5.5 min 40% B, 5.5–7.0 min 42%B, 7.0–9.0 min 50% B, 9.0–15.0 min 65% B, 15.0–17.0 min 75% B, 17.0–18.5 min 85% B, 18.5–19.5 min 95% B, 19.5–21 min 10% B, 21.0–25.0 min 10% B (prostaglandins and other oxylipins); and 0.0–2.0 min 30–45% B, 2.0–2.5 min 45–79% B, 2.5–11.5 min 79% B, 11.5–12 min 79–90% B, 12–14 min 90% B, 14–14.5 min 90–79% B, 14.5–15.5 min 79% B, 15.6–19 min 30% B (2-AG, AEA and related *N*-acylethanolamines).

Precursor ions, [M+H]^+^ and [M-H]^-^, product ions, multiple reaction monitoring (MRM) transitions and optimal collision energies were established for each analyte. ESI conditions were: capillary and nozzle voltage at 4000 V and 1500 V, drying gas temperature 230°C with a gas flow of 15 L/min, sheet gas temperature 400°C with a gas flow of 11 L/min, the nebulizer gas flow was 35 psi, and iFunnel high and low pressure RF at 90 and 60 V (negative mode) and 150 and 60 V (positive mode). The dynamic MRM option was performed for all compounds with optimized transitions and collision energies. The MassHunter Workstation software was used manually to integrate all peaks. The limits of quantification (LOQ) for compounds in the eCB metabolome were in the range 0.5–1000 fg on column, intraday accuracy and precision ranges (%) were 83–125 and 0.3–17, respectively, and interday accuracy and precision ranges (%) were 80–119 and 1.2–20, respectively, dependent upon the compound and the concentration studied. Corresponding values for the oxylipins were LOQ 0.5 fg– 4.2 pg on column (LOQ), 85–115% (inter- and intraday accuracy) and < 5% (precision) [24].

Internal standard recovery rates were established for RAW264.7 cells pellet methanolic extracts (5 replicates, test samples) and PBS (100 mM, 5 replicates). Briefly, samples were spiked with 10 μL of internal standard solutions and extracted by SPE as described above. To calculate recovery rates, internal standard calibration curves obtained at five different concentrations normalized against CUDA were used and expressed as the percentage of the expected value. Matrix-dependent recovery was established by spiking 10 μL internal standards in a similar manner to human plasma.

### Statistical analyses

pI_50_ and IC_50_ values were calculated using log(inhibitor) *vs*. response with variable slope (four parameters) algorithm in the GraphPad Prism computer program (GraphPad Software Inc., San Diego, CA. USA). The best fit was chosen by Akaike’s informative criteria. K_i_ values were obtained in two ways: 1) using the enzyme kinetics competitive model algorithm available in the GraphPad Prism programme; 2) from the intersection of the lines in a Dixon plot. The regression lines were determined by the robust analysis, rather than the least squares analysis, available in the GraphPad Prism programme. Oxygen consumption time courses were fitted to the”plateau followed by one phase delay” algorithms available in the GraphPad Prism programme. Kruskal-Wallis testing and post-hoc testing used Dunn’s multiple comparison test were undertaken using the same computer programme. The rank-based two-way ANOVAs (two-way robust Wilcoxon analysis [25]) were calculated using the function raov in the Rfit package of the R computer programme [26,27].

## Results

### Inhibition of COX isoforms *in vitro* by the enantiomers of Flu-AM1

**Fig 1:**
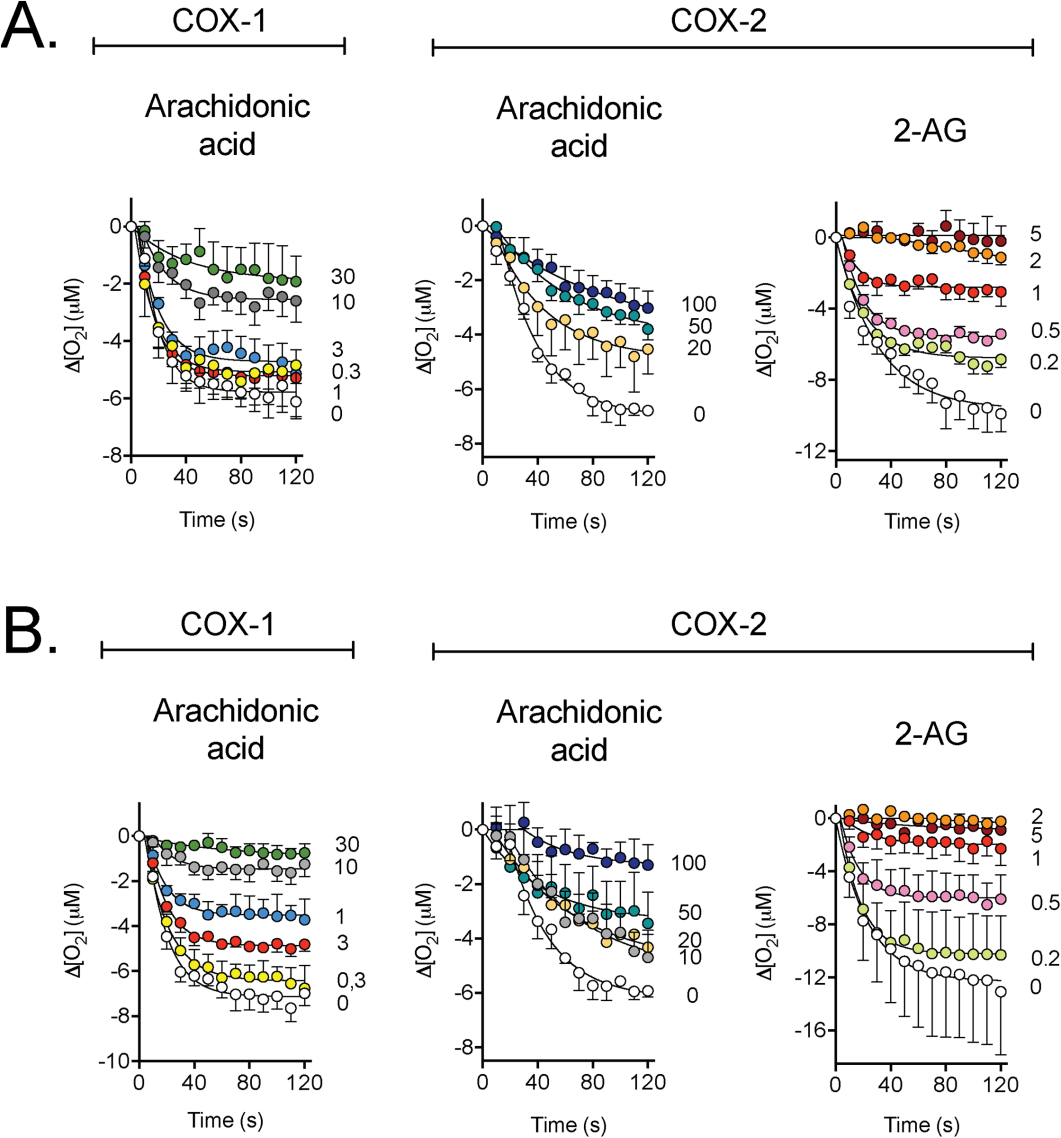
Inhibition of COX isoforms by the enantiomers of Flu-AM1. Shown are means ± s.e.m., n = 3 for the change in oxygen tension following addition of enzyme in the presence of the concentrations of Panel A, (*R*)-Flu-AM1 and Panel B, (*S*)-Flu-AM1. The concentrations, in μM, of the inhibitors are shown on the right of each panel, and the enzyme isoform and substrate (10 μM concentration) used above each panel.

The inhibition of ovine COX-1 and recombinant human COX-2 by the enantiomers of Flu-AM1 are shown in Fig 1. Both compounds were effective inhibitors of arachidonic acid oxidation by both isoform, and of 2-AG oxidation by COX-2. The curves in the figure were fitted to the built-in equation “plateau followed by one phase delay” in the GraphPad Prism programme, where the initial y value was set to zero and the x_o_ value (the length of the initial lag phase) was allowed to be in the range 0–120 s. From the mean values returned from the equation, initial values (at x_0_ + 1 s) were calculated and these were used to derive approximate IC_50_ values of: (*R*)-Flu-AM1, COX-1 (arachidonic acid) 6 μM; COX-2 (arachidonic acid) 20 μM; COX-2 (2-AG) 1 μM; (*S*)-Flu-AM1, COX-1 (arachidonic acid) 3 μM; COX-2 (arachidonic acid) 10 μM; COX-2 (2-AG) 0.7 μM. Thus, the (*S*)-enantiomer is roughly twice as potent as the (*R*)-enantiomer, but both enantiomers show substrate-selective inhibition of COX-2, whereby the oxygenation of 2-AG is inhibited at concentrations an order of magnitude lower than required for inhibition of the oxygenation of arachidonic acid.

**Fig 2:**
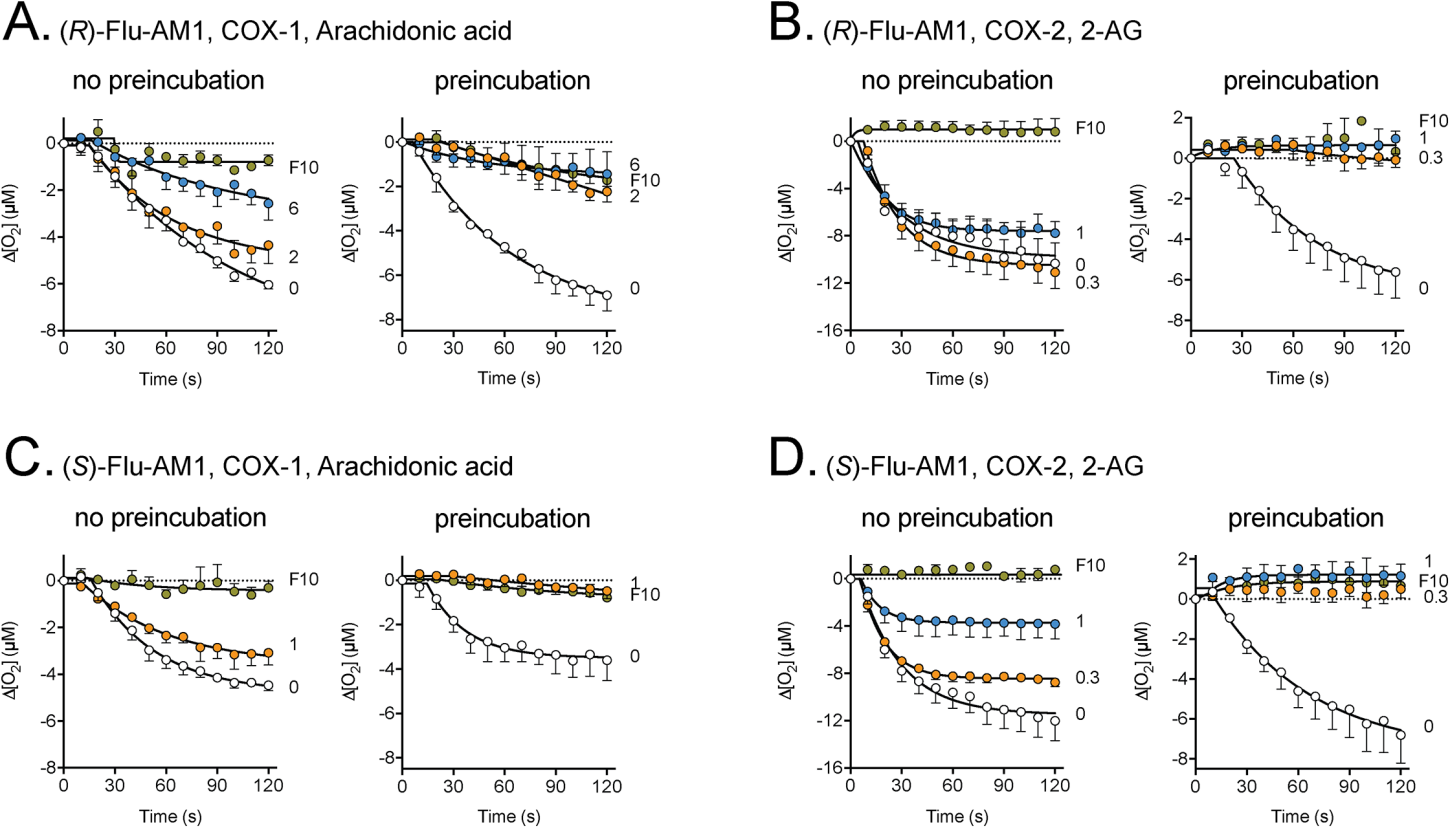
Effect of a preincubation phase on the inhibition of COX by the enantiomers of Flu-AM1. The concentrations, in μM, of the inhibitors are shown on the right of each panel (F10 refers to 10 μM racemic flurbiprofen, used as a positive control). The Flu-AM enantiomer, enzyme isoform and substrate (10 μM concentration) used is shown above each panel. The preincubation time was 5 min. Shown are means ± s.e.m., n = 3, except for the flurbiprofen curves in the no preincubation conditions in panels A and D, where n = 2.

Flurbiprofen inhibits COX in a time-dependent manner [28]. In order to determine whether the two enantiomers of Flu-AM1 also exhibited this property, the effect of sub-maximal concentrations of the compounds were investigated either without preincubation (where the reactions are started by addition of the enzyme) or following a five minute preincubation with enzyme prior to starting the reactions by addition of substrate. For both COX-1-catalysed oxygenation of arachidonic acid and COX-2-catalysed oxygenation of 2-AG, the inhibition was more prominent following the preincubation period (Fig 2).

### Inhibition of mouse brain FAAH by the enantiomers of Flu-AM1

**Fig 3:**
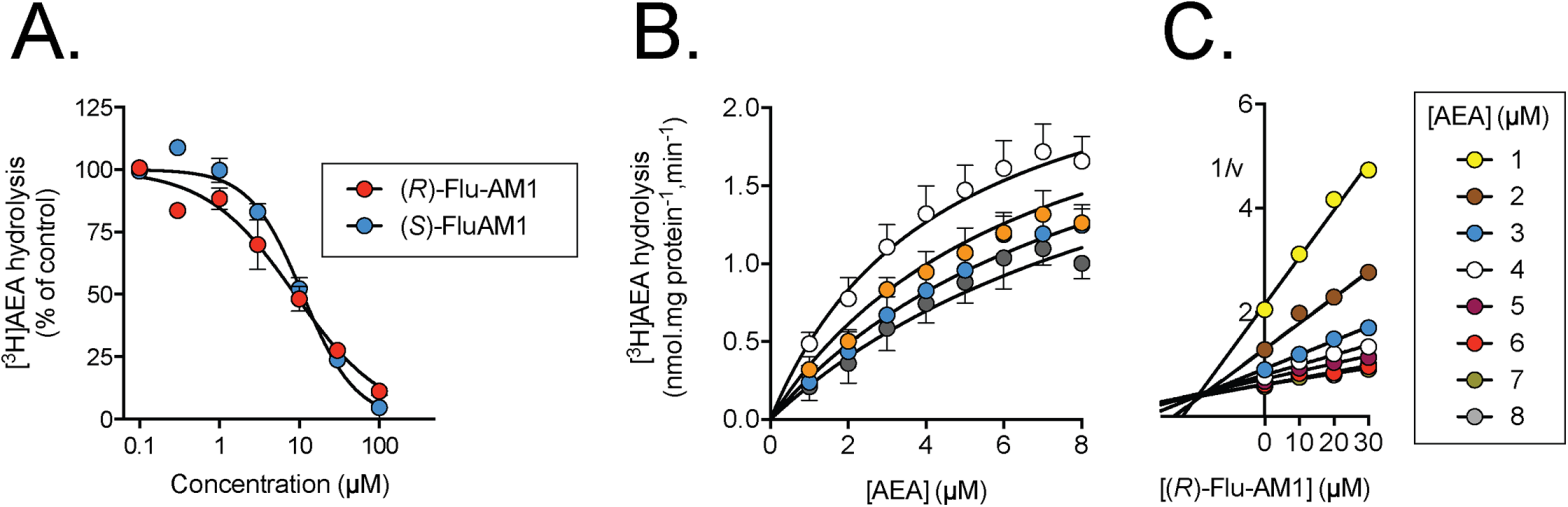
Inhibition of mouse brain FAAH by the enantiomers of Flu-AM1. Panel A. Concentration response curves for the inhibition of 0.5 μM [^3^H]AEA (means ± s.e.m., n = 3, when not enclosed by the symbols). Panel B. Kinetic experiments using 1–8 μM [^3^H]AEA (means ± s.e.m., n = 3, when not enclosed by the symbols). The curves were better fitted by a model assuming a competitive interaction (75% probability that it was correct, Akaike’s informative criteria) than a model assuming a mixed-type interaction (25% probability that it was correct, Akaike’s informative criteria). Panel C. Dixon replot of the mean data from Panel B. The intersection point of the regression lines projected onto the x-axis gives-K_i_.

The inhibition of [^3^H]AEA hydrolysis in mouse brain homogenates by the two enantiomers of Flu-AM1 is shown in Fig 3A. The potencies of the two enantiomers were very similar, with IC_50_ values of 8.8 and 11 μM for the (*R*)- and (*S*)-enantiomer, respectively. In kinetic experiments, (*R*)-Flu-AM1 behaved as a competitive inhibitor of FAAH with a K_i_ value of 20±8 μM (Fig 3B). A Dixon plot of the data gave the same K_i_ value (19 μM; Fig 3C). Further analysis of the Dixon plot for (*R*)-Flu-AM1 confirmed the assumption that, under the conditions used, the added AEA concentration is directly proportional to the free AEA concentration available to the enzyme (S1 Fig).

### Comparison of URB597, flurbiprofen, their combination and (*R*)-Flu-AM1 upon the lipid profile of RAW 264.7 cells

Treatment of RAW 264.7 cells, a mouse macrophage cell line, with bacterial lipopolysaccharide (LPS) results in the induction of COX-2 [29]. This cell line thus allows investigation of the effects of (*R*)-Flu-AM1 upon prostaglandin production in unstimulated and activated cells, and to determine whether COX-2-catalysed metabolism of 2-AG is a major metabolic route in the activated cells. Initial experiments indicated that the recovery rates of the lipids from RAW 264.7 cell extracts were very good (S2 Fig; see S1 Table for list of lipid abbreviations). Additionally, preliminary studies using LPS + INF-γ - treated RAW 264.7 cells were undertaken whereby all the medium from vehicle and ionomycin-treated conditions was collected, the lipids extracted and assayed. For the vehicle-treated cells, no lipid signal above the detection limit was observed in the medium extracts. For medium extracts from the ionomycin-treated cells, small peaks were seen for PEA, SEA, OEA, 13-HODE and 9(S)-HODE, but these were very close to the quantification limit. This would suggest that release of the lipids is limited under the conditions used here.

**Fig 4:**
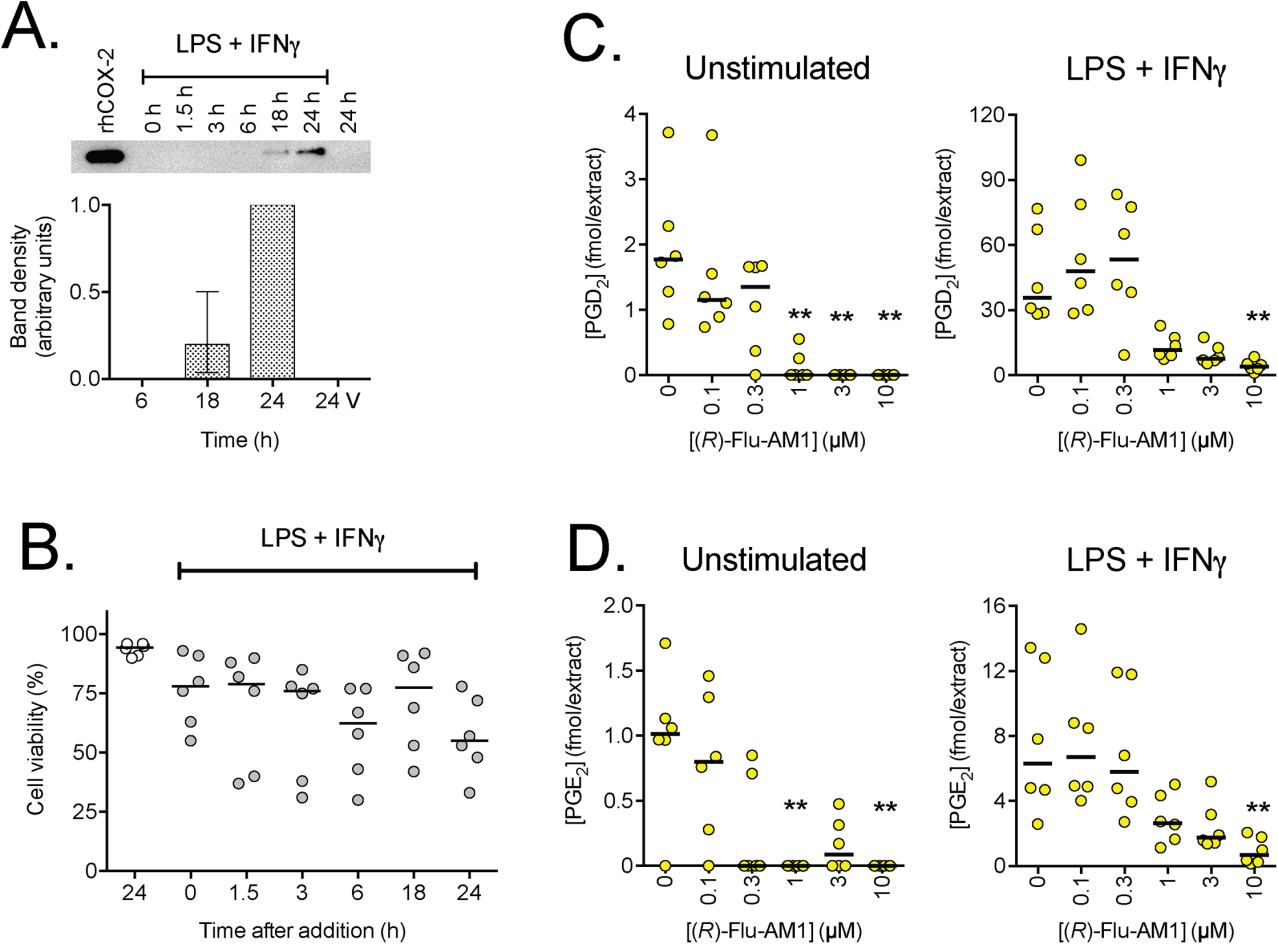
Effects of (*R*)-Flu-AM1 upon prostaglandin levels in basal and LPS + INF-γ-stimulated RAW 264.7 cells. Cells were either untreated (“unstimulated”) or treated for 24 h, unless otherwise shown, with 0.1 μg/mL LPS + 100 U/ml INF-γ. Panel A shows the expression of COX-2 at different treatment times. A representative gel is shown above the bar graph, where the intensities have been normalised to the values for 24 h (median and range, n = 3). rhCOX-2 refers to human recombinant COX-2 as a positive control. The bands shown in the gel were the only bands on the gels, and they were seen at a molecular weight of ~75 kDa. Panel B shows the viability of the cells at different times after treatment for different times (n = 6, with the bars showing the median values). In Panels C and D, the levels of PGD_2_ and PGE_2_, respectively, are shown (n = 6, expressed as fmol in the extract from two wells, with the bars showing the median values) following treatment with the compounds for 30 min. For both prostaglandins, two-way robust Wilcoxon analyses [25] indicated significant (P<0.001) effects of LPS + INF-γ treatment, of (*R*)-Flu-AM1, and of the interaction (*R*)-Flu-AM1 x LPS + INF-γ treatment. In view of the significant interaction, the curves shown in the figures were analysed separately for the unstimulated and stimulated cells. **P<0.01 (otherwise not significant) *vs*. vehicle-treated samples, Dunn’s multiple comparison test, following significant P value in the Kruskal-Wallis test.

The combination of 0.1 μg/mL of LPS + 100 U/mL of INF-γ produced the expected induction of COX-2 and robust increases in the levels of PGD_2_ and PGE_2_ following a 24 h incubation (Fig 4). There was a variable effect of the treatment upon cell viability (Fig 4B), so that the 24 h time-point used is a trade-off between the levels of COX-2 induction required for the study and effects upon cell viability. Increasing the LPS concentration to 1 μg/mL did not increase further the expressed level of COX-2, nor did increasing the incubation time to 48 h (data not shown).

**Fig 5:**
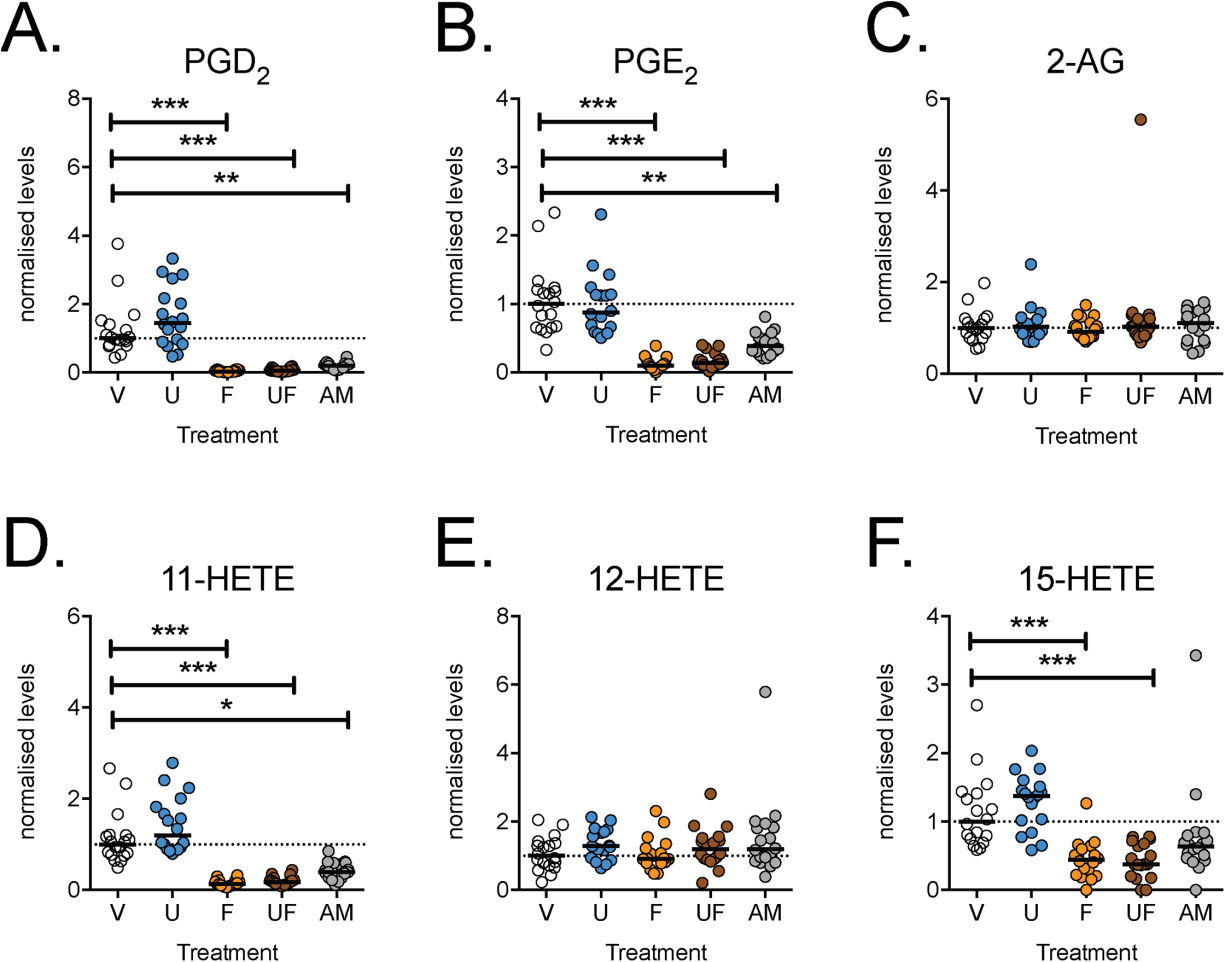
Effects of FAAH and/or COX inhibitors upon the levels of arachidonic acid-derivatives in ionomycin-treated RAW264.7 cells pretreated with LPS + IFNγ for 24 h. RAW264.7 mouse leukemic cells (2.5x10^5^ per well) were added to 6 well-plates with LPS (0.1 μg/mL well) and INF-γ (100 U/mL) and cultured at 37°C for 24 h prior to incubation for 30 min with ionomycin (5 μM) and either vehicle (V), URB597 (U, 1 μM) or (*R*)-Flu-AM1 (AM, 10 μM). The median value for each lipid and batch for vehicle-treated conditions were set to unity and all other values for the batch were expressed relative to these median values. Data are shown as scatter plots (n = 18), with the median values shown as bars. *P<0.05, **P<0.01, ***P<0.001, Dunn’s Multiple Comparison test *vs*. vehicle (otherwise not significant) following significant Kruskal-Wallis test.

Given that both enantiomers of Flu-AM1 had relatively similar properties towards COX (Figs 1 and 2), we focussed upon the (*R*)-enantiomer. An almost complete inhibition of both basal and LPS +INF-γ-stimulated PGD_2_ and PGE_2_ production was seen with the highest concentration of (*R*)-Flu-AM1 tested (10 μM), whereas more variable effects were seen with lower concentrations (Fig 4C and 4D). The effects of flurbiprofen (10 μM, either *per se* or together with 1 μM URB597) and (*R*)-Flu-AM1 (10μM) upon the levels of prostaglandins, 2-AG and related oxylipins were investigated in a series of experiments. For some of the lipids, not least the prostaglandins, there was a large variation in levels observed between batches, and so we have normalised the data to the corresponding vehicle controls. In the initial experiments, flurbiprofen and (*R*)-Flu-AM1 (10μM) blocked, as expected, both unstimulated and LPS + INF-γ - induced PGD_2_ and PGE_2_ in the cell extracts (Table 1). LPS + INF-γ treatment also increased 11-HETE and possibly 15-HETE levels in a manner sensitive to inhibition by flurbiprofen and (*R*)-Flu-AM1 (Table 1). This is consistent with the report that COX-2 in activated macrophages is capable of producing these oxylipins [30]. In contrast to the robust effects of flurbiprofen and (*R*)-Flu-AM1 upon prostaglandin levels in the cell extracts, the levels of 2-AG were not affected (Table 1). Similar results were seen in a larger series of LPS + INF-γ –treated cells where the calcium ionophore ionomycin was also added (Fig 5). Linoleic acid-derived oxylipins were also analysed, in order to shed light on possible off-targets for (*R*)-Flu-AM1. No significant effects of this compound upon the linoleic acid-derived oxylipins were seen (Table 1; S3 Fig).

**Table 1:** Lipid profile of RAW264.7 cells treated with or without LPS + IFNγ for 24 h. Effects of FAAH and/or COX inhibitors.

Lipid	Treatment	Vehicle	URB597	Flurbiprofen	URB + Flu	(*R*)FluAM1	P values
*Arachidonic acid derivatives*							
PGD_2_	None	1 (0.38–2.69)	1.37 (0.79–2.48)	0 (0–0.28)	0 (0–0)	0 (0–0.25)	**L: <0.0001**
[77; 2610]	LPS+ IFNγ	38.0 (12.0–69.6)	35.1 (19.4–117)	1.32 (1.04–4.76)	1.60 (1.22–3.72)	3.14 (1.51–4.13)	**I: <0.0001**
							**L x I: <0.0001**
PGE_2_	None	1 (0.46–1.58)	0.98 (0.49–1.81)	0 (0–0)	0.04 (0–0.23)	0 (0–0.25)	**L: <0.0001**
[22; 172]	LPS+ IFNγ	7.89 (2.10–20.4)	10.6 (7.33–33.5)	1.21 (0.91–4.54)	1.68 (0.64–2.36)	2.89 (1-28-3.30)	**I: <0.0001**
							**L x I: <0.0001**
11-HETE	None	1 (0.39–1.32)	1.12 (0–2.69)	0.67 (0.23–0.76)	0.83 (0–1.12)	0.55 (0.12–2.43)	**L: <0.0001**
[9.8; 112]	LPS+ IFNγ	11.8 (6.68–39.5)	13.0 (4.82–25.7)	2.06 (1.67–7.71)	2.52 (1.73–3.48)	2.29 (0.92–7.87)	**I: <0.0001**
							**L x I: <0.0001**
12-HETE	None	1 (0.22–1.42)	0.94 (0.33–1.48)	0.65 (0.46–2.01)	0.64 (0.58–1.18)	0.94 (0.27–1.82)	L: 0.020
[98; 62]	LPS+ IFNγ	0.59 (0.31–1.63)	0.61 (0.41–0.96)	0.43 (0.18–0.63)	0.78 (0.26–1.59)	0.32 (0.10–1.05)	I: 0.61
							L x I: 0.79
15-HETE	None	1 (0.71–1.58)	1.02 (0.25–2.16)	1.18 (0.15–1.85)	1.11 (0.72–2.06)	1.37 (0–2.72)	**L: 0.00011**
[23; 40]	LPS+ IFNγ	2.30 (1.20–8.30)	4.58 (0.97–5.29)	1.37 (0.51–8.43)	1.42 (0.97–1.75)	1.33 (0.67–3.37)	I: 0.035
							L x I: 0.020
AEA	None	1 (0.54–1.36)	0.85 (0.45–1.18)	0.74 (0.35–1.60)	0.99 (0.59–1.51)	0.65 (0.39–0.86)	**L: 0.00010**
[18; 33]	LPS+ IFNγ	1.15 (0.91–2.11)	1.39 (1.26–1.58)	1.17 (0.99–1.86)	1.19 (0.99–1.62)	1.01 (0.37–1.16)	I: 0.039
							L x I: 0.54
2-AG	None	1 (0.96–1.27)	1.10 (0.61–1.47)	1.10 (0.74–1.42)	1.09 (0.88–1.42)	0.94 (0.62–1.52)	**L: 0.00049**
[1980; 1600]	LPS+ IFNγ	0.83 (0.33–1.30)	0.90 (0.70–1.11)	0.65 (0.26–1.02)	1.03 (0.82–1.35)	0.81 (0.64–0.98)	I: 0.16
							L x I: 0.40
*Linoleic acid derivatives*							
9-HODE	None	1 (0.92–1.13)	1.14 (0.72–2.07)	1.12 (0.50–1.89)	1.41 (0.80–7.53)	0.99 (0.66–2.11)	L: 0.99
	LPS+ IFNγ	1.40 (0.70–2.74)	1.11 (0.49–1.20)	0.96 (0.62–8.38)	1.18 (0.74–1.54)	1.06 (0.50–1.64)	I: 0.67
							L x I: 0.78
13-HODE	None	1 (0.79–1.13)	1.42 (0.79–1.75)	1.19 (0.52–1.72)	1.57 (1.15–2.36)	0.97 (0.71–2.66)	L: 0.79
	LPS+ IFNγ	1.53 (0.81–2.94)	1.16 (0.49–1.43)	0.88 (0.76–7.61)	1.41 (1.10–1.92)	1.21 (0.55–1.75)	I: 0.38
							L x I: 0.54
9,10-DiHOME	None	1 (0.85–1.28)	1.10 (0.65–1.32)	0.89 (0.62–1.07)	0.99 (0.40–1.63)	1.05 (0.64–1.66)	L: 0.071
	LPS+ IFNγ	0.92 (0.85–1.04)	0.76 (0.69–1.05)	1.04 (0.64–1.32)	0.92 (0.72–1.01)	0.82 (0.65–1.09)	I: 0.93
							L x I: 0.10
12,13-DiHOME	None	1 (0.65–1.07)	1.15 (1.04–1.39)	0.89 (0.66–1.19)	1.32 (0.70–1.44)	1.12 (0.73–1.53)	L: 0.19
	LPS+ IFNγ	1.06 (0.90–1.21)	0.93 (0.85–1.26)	1.11 (0.64–2.30)	1.00 (0.79–1.35)	0.93 (0.76–1.04)	I: 0.39
							L x I: 0.034
9,10,13-TriHOME	None	1 (0.71–1.29)	1.13 (0.44–2.51)	1.20 (0.77–2.28)	1.41 (0.85–1.89)	1.04 (0.81–1.41)	L: 0.17
	LPS+ IFNγ	1.17 (0.69–1.44)	1.08 (0.68–1.89)	1.34 (0.80–1.40)	1.75 (1.13–2.09)	1.18 (0.90–1.49)	I: 0.0043
							L x I: 0.88
9,12,13-TriHOME	None	1 (0.69–1.32)	1.23 (0.51–2.53)	1.10 (0.90–1.93)	1.49 (0.98–1.77)	1.08 (0.76–1.57)	L: 0.37
	LPS+ IFNγ	1.15 (0.75–1.42)	1.08 (0.66–1.79)	1.33 (0.91–1.48)	1.65 (0.92–2.00)	1.22 (0.79–1.60)	I: 0.0089
							L x I: 0.85
13-oxo-ODE	None	1 (0.55–2.30)	1.40 (0.76–2.27)	1.33 (1.14–2.51)	1.53 (0.68–4.01)	1.97(0.59–3.00)	L: 0.19
	LPS+ IFNγ	1.92 (1.09–7.10)	1.52 (0.34–2.87)	2.14 (0.66–17.3)	1.72 (1.07–5.21)	1.49 (0.31–2.85)	I: 0.73
							L x I: 0.48
12(13)-EpOME	None	1 (0.27–1.61)	1.07 (0.66–1.66)	0.84 (0.75–1.81)	1.14 (0.78–8.48)	1.03 (0.51–5.45)	L: 0.60
	LPS+ IFNγ	1.03 (0.53–2.71)	1.24 (0.92–1.72)	0.99 (0.50–5.76)	1.40 (0.47–2.48)	1.02 (0.53–1.98)	I: 0.74
							L x I: 0.95
*Eicosapentaneoic acid derivative*							
12(*S*)-HEPE	None	1 (0.79–2.48)	0.59 (0–0.85)	0.44 (0.23–0.81)	0.48 (0.18–0.57)	0.90 (0.17–1.43)	L: 0.020
	LPS+ IFNγ	0.35 (0–0.89)	0.59 (0.28–1.36)	0.58 (0.14–0.82)	0.44 (0–0.62)	0.27 (0–0.74)	I: 0.16
							L x I: 0.011
*Other N-acyl ethanolamimes*							
PEA	None	1 (0.80–1.25)	1.07 (0.87–1.28)	0.94 (0.75–1.20)	1.06 (0.89–1.53)	0.89 (0.74–1.06)	L: 0.24
	LPS+ IFNγ	0.91 (0.56–1.26)	0.97 (0.88–1.31)	0.99 (0.53–1.12)	0.95 (0.65–1.16)	0.91 (0.49–1.15)	I: 0.39
							L x I: 0.87
SEA	None	1 (0.85–1.52)	0.80 (0.65–1.27)	0.83 (0.76–1.41)	1.11 (0.86–1.34)	0.90 (0.52–1.02)	L: 0.15
	LPS+ IFNγ	0.90 (0.65–1.25)	0.79 (0.67–1.28)	0.88 (0.62–1.10)	0.90 (0.74–1.09)	0.86 (0.65–1.11)	I: 0.046
							L x I: 0.36
OEA	None	1 (0.79–1.30)	0.94 (0-81-1.29)	0.87 (0.81–2.16)	1.14 (0.87–6.23)	1.01 (0.79–1.11)	L: 0.86
	LPS+ IFNγ	0.94 (0.80–1.22)	0.97 (0.74–1.96)	0.99 (0.86–1.43)	1.11 (0.91–1.23)	1.03 (0.87–1.22)	I: 0.19
							L x I: 0.69
LEA	None	1 (0.81–1.81)	0.79 (0.68–1.15)	1.01 (0.72–1.99)	1.15 (0.94–12.8)	1.07 (0.21–1.26)	L: 0.84
	LPS+ IFNγ	0.96 (0.69–1.09)	1.06 (0.79–1.87)	0.91 (0.32–1.59)	1.11 (0.83–1.66)	1.13 (0.33–1.60)	I: 0.43
							L x I: 0.49

RAW264 mouse leukemic cells (2.5x10^5^ per well) were added to 6 well-plates and incubated with either vehicle (“None”) or LPS (0.1 μg/mL well) + INF-γ (100 U/mL) for 24 h. at 37°C prior to incubation for 30 min with either vehicle, URB597 (1 μM), flurbiprofen (10 μM), URB597 + flurbiprofen or (*R*)-Flu-AM1 (10 μM). The samples were assayed in two batches. In order to minimise effects of inter-batch variations, which were seen for some of the lipids, the median value for each lipid and batch under vehicle-treated “None” conditions were set to unity and all other values for the batch were expressed relative to these median values. Data are given as medians, n = 5–6, with the range in brackets, and the statistical test used was a two-way robust Wilcoxon analysis [25]. L: main effect LPS treatment, I: main effect inhibitor treatment, T x I, interaction term. Note that since there are multiple analysis, a case can be made that the Bonferroni correction should be used. Significance levels below 0.0025 (= 0.05/20, i.e. Bonferroni-corrected) are shown in bold.

**Fig 6:**
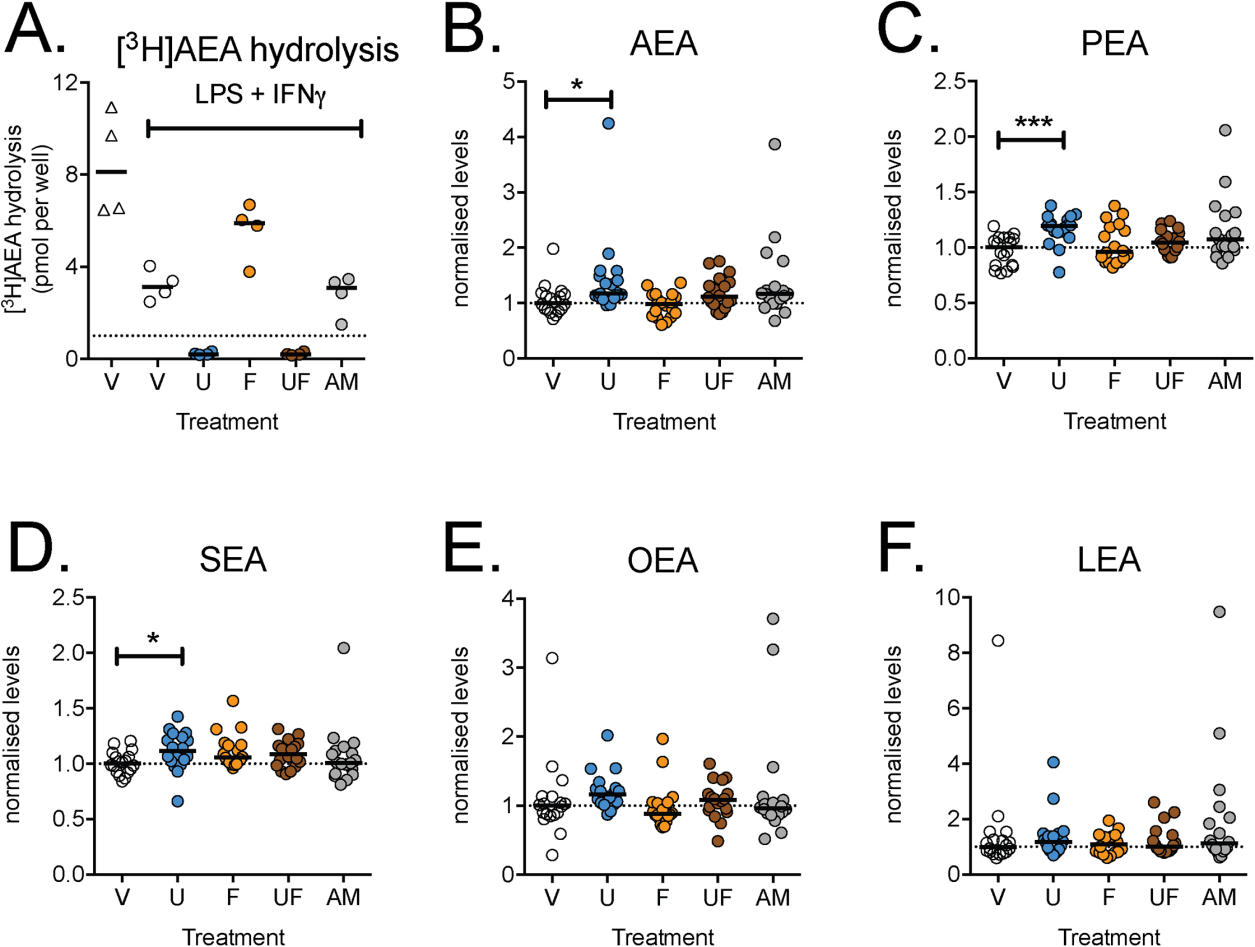
Effects of FAAH and/or COX inhibitors upon A. the hydrolysis of exogenously added [^3^H]AEA and B-F the levels of AEA and related *N*-acylethanolamines in RAW 264.7 cells. In Panel A, 2.5 x 10^6^ cells/well were seeded and cultured overnight prior to addition of either phosphate-buffered saline or LPS (0.1 μg/mL) + INF-γ (100 U/mL) and incubation for a further 24 h. The hydrolysis of 100 μM [^3^H]AEA is shown for the different treatments (following 1 h of incubation. In Panels B-F, cells (2.5x10^5^ per well) were added to 6 well-plates with LPS (0.1 μg/mL well) and INF-γ (100 U/mL) and cultured at 37°C for 24 h. prior to incubation for 30 min with ionomycin (5 μM) and either vehicle (V), URB597 (U, 1 μM) or (*R*)-Flu-AM1 (AM, 10 μM). The median value for each lipid and batch for vehicle-treated conditions were set to unity and all other values for the batch were expressed relative to these median values. Data are shown as scatterplots (n = 18) with medians as bars. *P<0.05,***P<0.001, Dunn’s Multiple Comparison test *vs*. vehicle (otherwise not significant) following significant Kruskal-Wallis test.

To determine the ability of LPS + INF-γ –treated RAW 264.7 cells to hydrolyse exogenous [^3^H]AEA (100 nM), the cells were incubated with this substrate for 60 min in the absence or presence of the compounds. As expected, 1 μM URB597, either *per se* or with flurbiprofen, completely blocked [^3^H]AEA hydrolysis (Fig 6A). Given the potencies of flurbiprofen and (*R*)-Flu-AM1 towards mouse FAAH are modest, clear effects of these compounds at the concentration of 10 μM upon [^3^H]AEA hydrolysis by the intact mouse RAW 264.7 cells would not be expected, and this was found to be the case (Fig 6A). Surprisingly, however, URB597 only produced modest effects upon the levels of AEA and related *N*-acylethanolamines in the cells (Table 1, Fig 6B–6H). Thus, at a concentration of URB597 causing complete inhibition of the hydrolysis of exogenous AEA, endogenous levels are only marginally affected. Flurbiprofen and (*R*)-Flu-AM1 did not affect the levels of these lipids in the ionomycin treated cells (Fig 6).

## Discussion

In the present study, the enantiomers of Flu-AM1 were investigated in order to shed light on two questions that were asked at the end of the introduction. These questions are recapitulated below, to aid the discussion:

### Do the amide derivatives of flurbiprofen with 2-amino-3-methylpyridine show enantiomeric differences with respect to inhibition of COX-2 and FAAH?

This question was motivated by previous studies showing that (*R*)-profens (ibuprofen, flurbiprofen, naproxen) retained the substrate-selective inhibition of COX-2 seen in the enantiomers, but lacked significant effect upon arachidonic acid oxygenation by either COX isoform [11]. Mutagenesis and computer modelling approaches have been very informative in elucidating the interaction of NSAIDs with both COX and FAAH [31,32]. The interaction of profen enantiomers with COX has been studied using both approaches [11,33], and crystallographic studies have suggested that a critical interaction for the (*R*)-profens is the ability of the carboxyl group to ion pair with the Arg^120^ residue in COX-2 [11], whilst molecular modeling of the binding of (*R*)-flurbiprofen suggests that the phenolic group of Tyr^355^ would interact with the α-methyl group of this inhibitor so as to interfere with the binding of the carboxylate group to Arg^120^, thereby accounting for the poor potencies of the (*R*)-enantiomers towards the oxygenation of arachidonic acid [33]. The two enantiomers of Flu-AM1 retain the time-dependency of COX inhibition seen with flurbiprofen [28] but do not show marked differences in their COX-inhibitory properties. This latter finding presumably reflects the fact that they contain an uncharged amide group instead of the negatively charged carboxyl group of flurbiprofen and suggests interaction with COX different from ion pair formation with the Arg^120^ residue. It would clearly be of interest to investigate using computational and mutagenesis techniques the interaction of the compounds with COX isoforms, and, indeed, with FAAH.

The two compounds show a degree of substrate-selectivity towards the inhibition of 2-AG oxygenation by COX-2 *vs*. arachidonic acid oxygenation by this isoform. For comparative purposes, recalculation by the method used here of data for racemic flurbiprofen obtained using the same method [18] gave IC_50_ values for this NSAID of: COX-1 (arachidonic acid) 4 μM; COX-2 (arachidonic acid) 95 μM; COX-2 (2-AG) 2 μM. Thus, the compounds are approximately equipotent to flurbiprofen as inhibitors of COX-1, but more potent inhibitors of 2-AG oxygenation by COX-2. The enantiomers of Flu-AM1 showed very similar potencies towards inhibition of mouse brain FAAH, a result also seen for flurbiprofen enantiomers and rat brain FAAH [34]. It was noted that the potencies were lower than previously reported for racemic Flu-AM1 (IC_50_ value 0.44 μM [18]). This seems to reflect a species difference, since the racemate was studied in rat brain homogenates, whereas mouse brain homogenates were used here. Indeed, in rat brain homogenates, (*R*)- and (*S*)-Flu-AM1 inhibit [^3^H]AEA hydrolysis with IC_50_ values of 0.74 and 0.99 μM, respectively (current authors, unpublished data). We have elected to present the mouse data here, since the lipidomic work described below was conducted on RAW264.7 cells, which are murine in origin.

### Is COX-2 inhibition sufficient to affect endocannabinoid levels in macrophage cells cultured under inflammatory conditions?

Duggan et al. [11] reported that in primary cultures of mouse dorsal root ganglia cells stimulated with granulocyte-macrophage colony-stimulating factor followed by LPS, IRN-γ and 15(*S*)-HETE, resulting in the induction of COX-2, the inhibition of AEA and 2-AG oxygenation by (*R*)-profens increased the levels of these eCBs in the cell extracts, without affecting arachidonic acid levels. This would suggest that in these cells (which lack FAAH [11], in contrast to the situation for the dorsal root ganglia *in vivo* [35]), COX-2 is an important determinant of eCB metabolism. We found that at concentrations of 10 μM, both flurbiprofen and (*R*)-Flu-AM1 completely blocked prostaglandin production by both unstimulated and LPS + IFN-γ-treated RAW 264.7 macrophage cells, indicating that under these conditions the compounds block arachidonic acid oxygenation by both COX isoforms. However, this blockade did not affect the observed levels of either 2-AG or AEA. Thus, COX-2 appears to play a minor role in gating the catabolism of these eCBs in the RAW 264.7 cells, in contrast to the stimulated primary cultures of mouse dorsal root ganglia cells [11].

The present study has allowed us to answer an additional question: does FAAH inhibition affect endocannabinoid levels in macrophage cells cultured under inflammatory conditions? We found that URB597 produces significant, but rather small changes in the levels of AEA and related *N*-acylethanolamines that are FAAH substrates in the LPS + IFN-γ-treated RAW 264.7 cells despite the essentially complete inhibition of the hydrolysis of exogenously added [^3^H]AEA at the concentration of the compound used (1 μM). There are two explanations for this finding. It is possible that in the LPS + IFN-γ-treated RAW 264.7 cells, the turnover of the *N*-acylethanolamines is so slow that blockade of FAAH produces little effect. This would be the case, for example, if the synthetic pathways were the rate-limiting step in the life cycle of these lipids. There is evidence in the literature that LPS treatment increases the rate of AEA synthesis and concentration in RAW 264.7 cells despite a reduction in the expression at the mRNA level of the *N*-acylethanolamine synthetic enzyme *N*-arachidonoyl phosphatidylethanolamine-phospholipase D [36–38]. The primary pathway for AEA synthesis in the cells was instead identified as the production and then dephosphorylation of phospho-AEA [37]. In our hands, we found a modest, albeit significant, increase in AEA, but not the other *N*-acylethanolamines, levels following LPS + IFN-γ-treatment (Table 1). It is possible that under the conditions used here, the phospho-AEA pathway is less active than in the study of Liu et al. [37], and this results in the synthesis rather than hydrolysis being rate-limiting, even following ionomycin treatment.

An alternative (or additional) explanation is that the metabolism of endogenous AEA in the cells is less dependent upon FAAH than the hydrolysis of exogenously added AEA and that other catabolic enzymes are of greater importance. Given that the combination of flurbiprofen + URB597 did not affect levels of AEA in the RAW 264.7 cells, COX-2 can be ruled out as a candidate. The most likely enzyme is NAAA, given that it is highly expressed in macrophages [39]. NAAA inhibitors are beginning to appear in the literature, and one of these, 1-(2-biphenyl-4-yl)ethyl-carbonyl pyrrolidine, has been reported to restore PEA levels that were decreased in LPS-treated RAW 264.7 cells [40] Hopefully, more data will emerge on the effects of NAAA inhibitors on AEA as well as PEA levels in RAW 264.7 cells in the future.

## Conclusions

There are two main conclusions to the present study. Firstly, we find that in contrast to the profens, the two enantiomers of Flu-AM1 show little difference with respect to their ability to inhibit COX isoforms. The compounds also inhibit FAAH with similar potencies. Thus, there is little advantage in using one or other of the enantiomers over using the racemate. Secondly, our data show that in activated RAW 264.7 cells, COX-2 plays a relatively minor role in regulating eCB levels, and that the importance of FAAH for the hydrolysis of endogenous AEA may be less pronounced than for exogenously added AEA.

## Supporting Information

S1 Fig Estimated relationship between the added AEA concentration and the free substrate concentration available to the enzyme. Values are calculated from the data shown in Fig 2C as follows: for the general case, a linear mixed-type inhibition, the intersection point in the Dixon plot projected onto the y-axis (“y_i_”) corresponds to (1/V_max_)/(1-(1/α)) (Segel, 1975; α→∞ for competitive inhibition). Since, in the absence of inhibitor, the observed velocity v_o_ = V_max_/(1 + K_m_/[S_f_]), where [S_f_] in this case is the free AEA concentration presented to the enzyme, the two equations can be used to express [S_f_]/K_m_ in terms of the observed velocity, α and y_i_: [S_f_]/K_m_ = y_i_/[(1/v_o_)(1-(1/α))—y_i_]. This has been used here to generate a plot of different values of [S_f_]/K_m_ *vs*. the added AEA concentration for different given values of α. When (1/v)(1-(1/α)) ≈ y_i_, very small differences in y_i_ have a large effect on [S_f_]/K_m_, and so we have limited the lowest value of α in the graph to 15. The data indicate that under the conditions used, the added AEA concentration is proportional to the free AEA concentration presented to the enzyme.
(TIF)

S2 Fig Recovery rates of lipids used as internal standards spiked to RAW 264.7 cell extracts and to phosphate-buffered saline (PBS). Internal standard recovery rates were determined as described in Materials and Methods. Shown are means ± s.e.m. for five determinations.
(TIF)

S3 Fig Effects of FAAH and/or COX inhibitors upon the levels of linoleic acid-derivatives in ionomycin-treated RAW264.7 cells pretreated with LPS + IFNγ for 24 h. RAW264.7 mouse leukemic cells (2.5x10^5^ per well) were added to 6 well-plates with LPS (0.1 μg/mL well) and INF-γ (100 U/mL) and cultured at 37°C for 24 h. prior to incubation for 30 min with ionomycin (5 μM) and either vehicle (V), URB597 (U, 1 μM) or (*R*)-Flu-AM1 (AM, 10 μM). The median value for each lipid and batch for vehicle-treated conditions were set to unity and all other values for the batch were expressed relative to these median values. Data are shown as scatter plots (n = 18), with the median values shown as bars. *P<0.05, Dunn’s Multiple Comparison test *vs*. vehicle (otherwise not significant) following significant Kruskal-Wallis test.
(TIF)

S1 Table List of abbreviations of the lipids reported for the RAW264.7 cells. (DOCX)
