# Supplementary figures and images for: Characterisation of (R)-2-(2-Fluorobiphenyl-4-yl)-N-(3-Methylpyridin-2-yl)Propanamide as a Dual Fatty Acid Amide Hydrolase: Cyclooxygenase Inhibitor
Source: PLoS One. 2015 Sep 25;10(9):e0139212. doi: 10.1371/journal.pone.0139212 (PMC4583449; doi:10.1371/journal.pone.0139212)

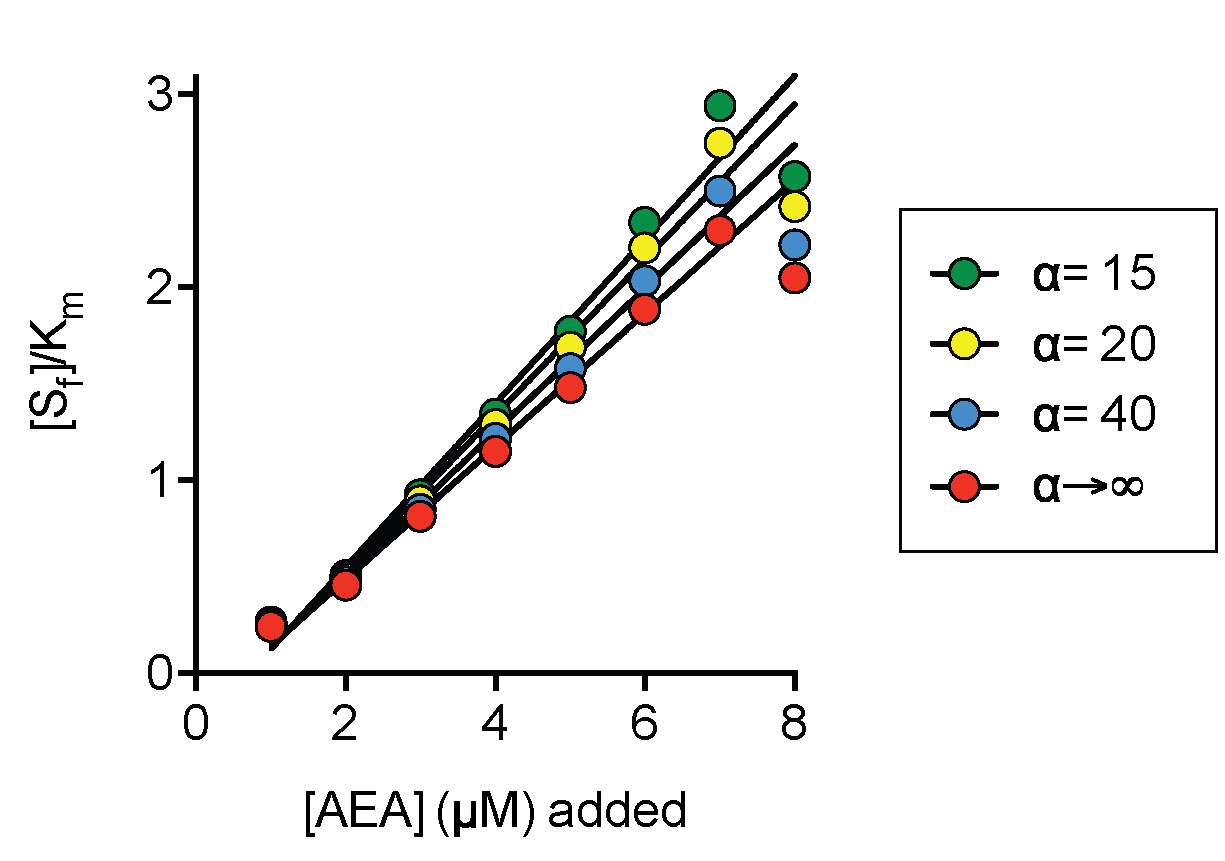

Supplement: S1 Fig — Values are calculated from the data shown in Fig 2C as follows: for the general case, a linear mixed-type inhibition, the intersection point in the Dixon plot projected onto the y-axis (“yi”) corresponds to (1/Vmax)/(1-(1/α)) (Segel, 1975; α→∞ for competitive inhibition). Since, in the absence of inhibitor, the observed velocity vo = Vmax/(1 + Km/[Sf]), where [Sf] in this case is the free AEA concentration presented to the enzyme, the two equations can be used to express [Sf]/Km in terms of the observed velocity, α and yi: [Sf]/Km = yi/[(1/vo)(1-(1/α))—yi]. This has been used here to generate a plot of different values of [Sf]/Km vs. the added AEA concentration for different given values of α. When (1/v)(1-(1/α)) ≈ yi, very small differences in yi have a large effect on [Sf]/Km, and so we have limited the lowest value of α in the graph to 15. The data indicate that under the conditions used, the added AEA concentration is proportional to the free AEA concentration presented to the enzyme. (TIF) [file pone.0139212.s001.tif]

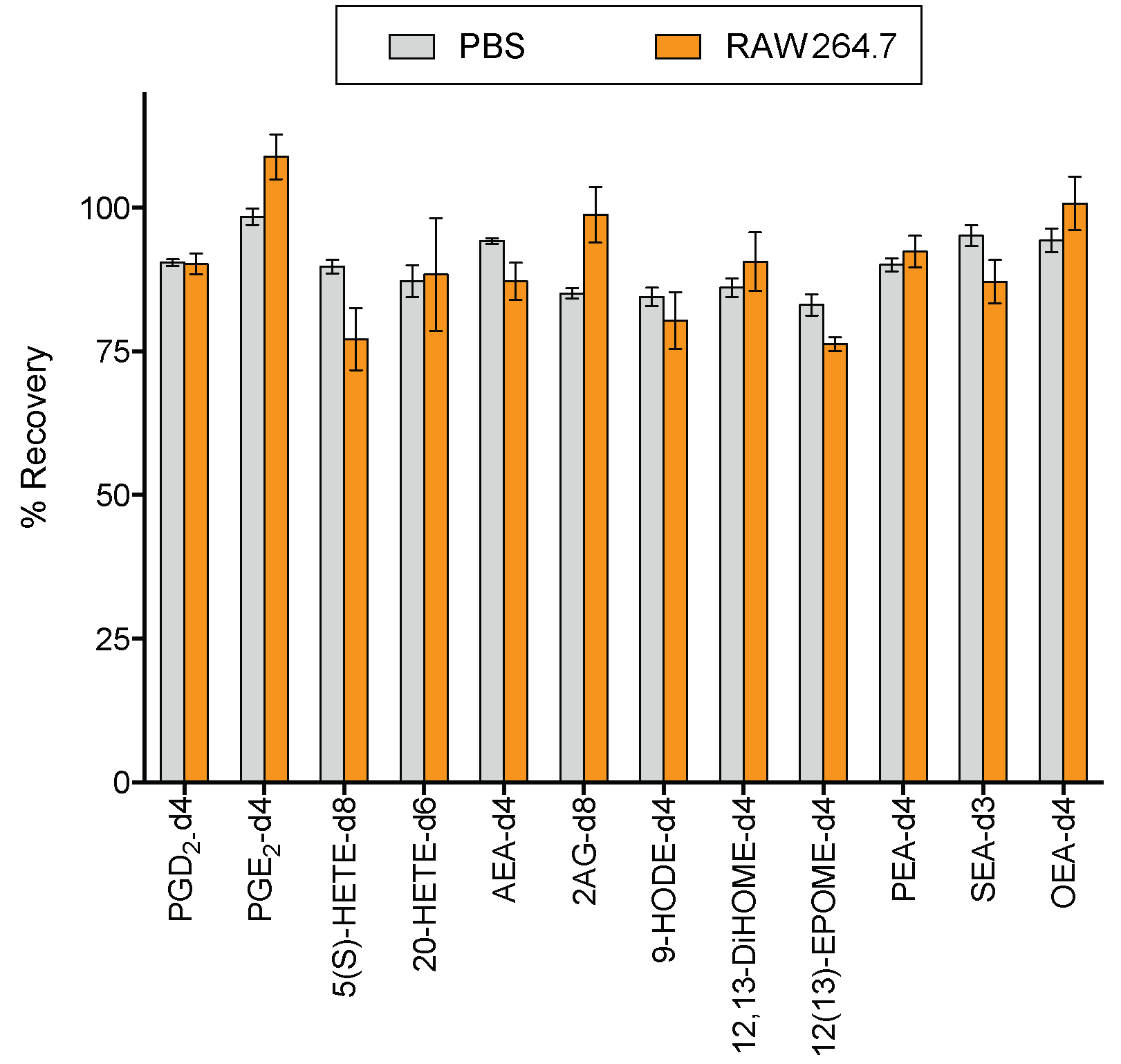

Supplement: S2 Fig — Internal standard recovery rates were determined as described in Materials and Methods. Shown are means ± s.e.m. for five determinations. (TIF) [file pone.0139212.s002.tif]

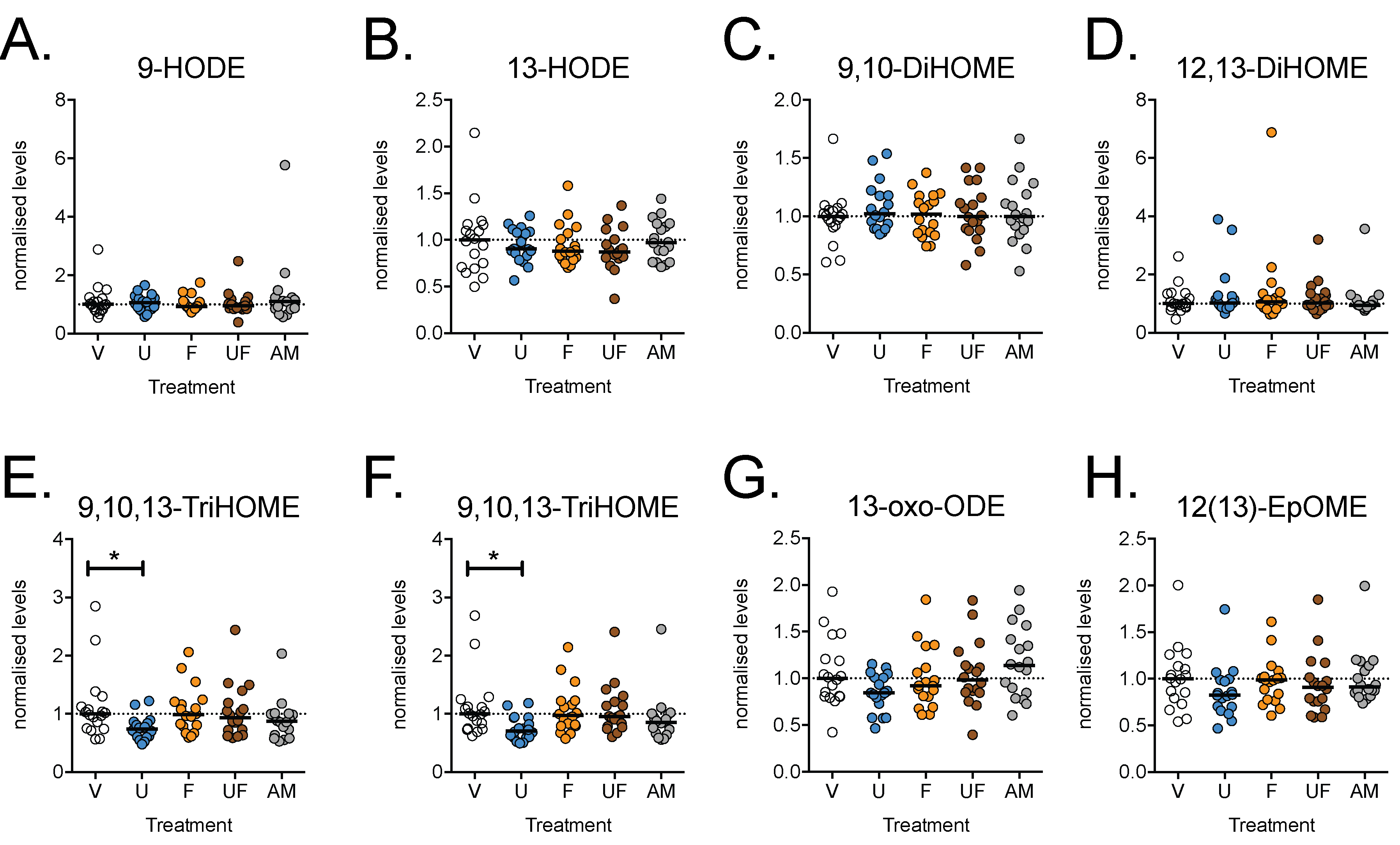

Supplement: S3 Fig — RAW264.7 mouse leukemic cells (2.5x10^5^ per well) were added to 6 well-plates with LPS (0.1 μg/mL well) and INF-γ (100 U/mL) and cultured at 37°C for 24 h. prior to incubation for 30 min with ionomycin (5 μM) and either vehicle (V), URB597 (U, 1 μM) or (R)-Flu-AM1 (AM, 10 μM). The median value for each lipid and batch for vehicle-treated conditions were set to unity and all other values for the batch were expressed relative to these median values. Data are shown as scatter plots (n = 18), with the median values shown as bars. *P<0.05, Dunn’s Multiple Comparison test vs. vehicle (otherwise not significant) following significant Kruskal-Wallis test. (TIF) [file pone.0139212.s003.tif]
