# Supplementary material for: Characterisation of (R)-2-(2-Fluorobiphenyl-4-yl)-N-(3-Methylpyridin-2-yl)Propanamide as a Dual Fatty Acid Amide Hydrolase: Cyclooxygenase Inhibitor
Source: PLoS One. 2015 Sep 25;10(9):e0139212. doi: 10.1371/journal.pone.0139212 (PMC4583449; doi:10.1371/journal.pone.0139212)
Supplement: S1 Table — (DOCX) [file pone.0139212.s004.docx]

**Supporting Information S1 Table. List of abbreviations of the lipids reported for the RAW264.7 cells**

|  |  |
| --- | --- |
| Abbreviation | Full name |
|  | |
|  | |
| *Arachidonic acid derivatives* | |
| PGD_2_ | 9α, 15(*S*)-dihydroxy-11-oxo-prosta-5*Z*,13*E*-dien-1-oic acid, prostaglandin D_2_ |
| PGE_2_ | 9-oxo-11α,15(*S*)-dihydroxy-prosta-5*Z*,13*E*-dien-1-oic acid, prostaglandin E_2_ |
| PGF_2α_ | 9α,11α,15(S)-trihydroxyprosta-5Z,13E-dien-1-oic acid, prostaglandin F_2α_ |
| 11-HETE | 11-hydroxy-5*Z*,8*Z*,12*E*,14*Z*-eicosatetraenoic acid |
| 12-HETE | 12-hydroxy-5*Z*,8*Z*,10*E*,14*Z*-eicosatetraenoic acid |
| 15-HETE | 15-hydroxy-5*Z*,8*Z*,11*Z*,13*E*-eicosatetraenoic acid |
| AEA | *N*-(2-hydroxyethyl)-5*Z*,8*Z*,11*Z*,14*Z*-eicosatetraenamide, arachidonoylethanolamide, anandamide |
| 2-AG | 5*Z*,8*Z*,11*Z*,14*Z*-eicosatetraenoic acid, 2-glyceryl ester, 2-arachidonoylglycerol |
|  | |
| *Linoleic acid derivatives* | |
| 9(*S*)-HODE | 9(*S*)-hydroxy-10*E*,12*Z*-octadecadienoic acid |
| 13-HODE | 13-hydroxy-10*E*,12*Z*-octadecadienoic acid |
| 9,10-DiHOME | 9(10)-dihydroxy-12*Z*-octadecenoic acid |
| 12,13-DiHOME | 12(13)-dihydroxy-9*Z*-octadecenoic acid |
| 9,10,13-TriHOME | 9,12,13-trihydroxy-11-octadecenoic acid |
| 9,12,13-TriHOME | 9,12,13-trihydroxy-10*E*-octadecenoic acid |
| 13-oxo-ODE | 12-oxo-9*Z*,11*E*- octadecadienoic acid |
| 12(13)-EpOME | 12(13)epoxy-9*Z*-octadecenoic acid, *iso*-leukotoxin |
|  |  |
| *Eicosapentaenoic acid derivative* | |
| 12(*S*)-HEPE | 12(*S*)-hydroxy-5*Z*,8*Z*,10*E*,14*Z*,17*Z*-eicosapentaenoic acid |
|  |  |
| *Other N-acyl ethanolamimes* | |
| LEA | *N*-(2-hydroxyethyl)-9*Z*,12*Z*-octadecadienamide, linoleoylethanolamide |
| PEA | *N*-(2-hydroxyethyl)-hexadecanamide, palmitoylethanolamide, palmidrol |
| SEA | *N*-(2-hydroxyethyl)-octadecanamide, stearoylethanolamide |
| OEA | *N*-(2-hydroxyethyl)-9*Z*-octadecenamide, oleoylethanolamide |
|  |  |
